# Survival Benefit of Metformin Use for Pancreatic Cancer Patients Who Underwent Pancreatectomy: Results From a Meta-Analysis

**DOI:** 10.3389/fmed.2020.00282

**Published:** 2020-07-30

**Authors:** Junqiang Zhang, Jichun Ma, Lingyun Guo, Bo Yuan, Zuoyi Jiao, Yumin Li

**Affiliations:** Department of General Surgery, Lanzhou University Second Hospital, Lanzhou, China

**Keywords:** pancreatic cancer, metformin, pancreatectomy, overall survival, meta-analysis

## Abstract

**Objective:** To evaluate the survival benefit of metformin use for pancreatic cancer (PC) patients underwent pancreatectomy.

**Methods:** Databases including EMBASE, PubMed, the Cochrane Library were searched to identify studies relevant to the outcomes on the survival benefit of metformin use for the PC patients who underwent pancreatectomy until June 30, 2019. STATA 12.0 software was used to performed the meta-analysis.

**Results:** 12 studies involving 35,346 PC patients were included in this meta-analysis. With a random-model, there are significant differences in overall survival (HR = 0.85, 95% CI: 0.77–0.94, *P* = 0.002) between PC patients who were treated with metformin underwent pancreatectomy and those who underwent pancreatectomy without metformin use. Subgroup analyses showed Caucasians (HR = 0.903, 95% CI = 0.825–0.940, *P* = 0.008) and Asian (HR = 0.691, 95% CI = 0.588–0.813, *P* = 0.001) PC patients have a significantly reduced risk of death for metformin users. Subgroup analyses also showed a survival benefit for PC patients at stage I-II (HR = 0.762, 95% CI = 0.677–0.858, *P* = 0.0001).

**Conclusions:** Metformin use is related to a better survival benefit for PC patients who underwent pancreatectomy, which would be a potential drug for the treatment of PC.

## Background

Pancreatic cancer, reported as the 4th death-leading cause worldwide (1) and was predicted to be the second death-leading cause by 2030. For diagnosis of PC, many of them were diagnosed at an unresectable stage or a distant metastasis stage (2). However, the patients with PC had a lower survival rate. The research reported that <20% of PC patients benefit from current surgery and the rate of 5-year survival is not even higher than 5% (3). Despite this, curative resection has improved the survival outcomes for PC patients over decades and tumor further progression and recurrence are still influenced by the great variability of chemotherapeutics resistance and clinical responses even for some patients with appropriate surgery or at early tumor stage (4, 5), which highlights that it is necessary for us to find better treatment strategies and survival risk factors for the patients with PC (6, 7).

Recently, a growing number of evidences suggested that anti-diabetic drug metformin can inhibit the division of cancer cells, down-regulate the level of circulating insulin and activate the immune-system for cancer patients (8). In addition, some hypoglycemic drugs can enhance the therapeutic outcomes by effecting the metabolic pathway with a result of inhibiting the malignant tumor cells, and also can control the blood glucose for individuals (9). Of which, metformin is the most promising adjuvant for cancer therapy (10). Although it was repeatedly reported that metformin plays an important role in decreasing the mortality and incidence of PC by epidemiologic and basic researches, the survival benefit of metformin use for PC patients who underwent pancreatectomy is still unclear. Therefore, we conducted a meta-analysis to evaluate the survival benefit of metformin use for patients with PC who underwent pancreatectomy.

## Materials and Methods

### Databases Searching

To include studies about the survival benefit of metformin use for patients with pancreatic cancer who underwent pancreatectomy, a comprehensive databases search including PubMed, the Cochrane Library and EMBASE was performed until June 30, 2019. Literature search terms were as follows: “pancreatic cancer,” “Pancreatic Neoplasm,” “Pancreas cancer,” “Pancreatic Ductal Carcinomas,” “PC,” “metformin,” “overall survival.” The language of the included study was limited to English in this meta-analysis.

### Selection Criteria

Selection criteria were listed as follows: (1) the pancreatic cancer was diagnosed by histological or pathological examination; (2) PC patients were treated with pancreatectomy surgery. (3) survival outcomes including overall survival were reported in full text; (4) survival outcomes were reported on hazard ratio (HR) and its 95% confidence interval (95% CI), and (5) the full research was published in English.

The articles including the following were excluded: (1) duplicate studies; (2) conference abstracts, case report, editorial letters and review; (3) without full text; (4) survival outcomes were not reported in full articles.

### Data Extraction

The relevant data was extracted from included studies by two reviewers (ZJQ and MJC) independently. Data retrieved from included studies as follows: (1) characteristics of studies including publications, authors, year of publication, sample size, country, pancreatectomy strategy, duration of follow-up; (2) clinical outcome: the data of overall survival.

### Quality Assessment

The quality of included studies was assessed according to the Newcastle-Ottawa scale (NOS) (11): (1) the selection of cohorts (0–4 points); (2) comparability of cohorts (0–2 points); (3) the exposure or outcome of the participant (0–3 points). Finally, the total score of each study represented the overall result of quality assessment. Studies with 7–9 points were regarded as “high quality.”

### Data Analysis

STATA version 12.0 was performed to process all data for this meta-analysis. The heterogeneity between included studies which were evaluated by using *I*^2^-based Q-test: if *p*-value was higher than 0.1 or *I*^2^ was lower than 50%, fixed effect model was used to pool the HR and its 95%CI. If not, the random effect model was adopted. Subgroup analyses were performed according to ethnicity and tumor clinical stage. Funnel plots were used to measure the bias of potential publication.

## Results

### Characteristics of Included Studies

**Figure 1:**
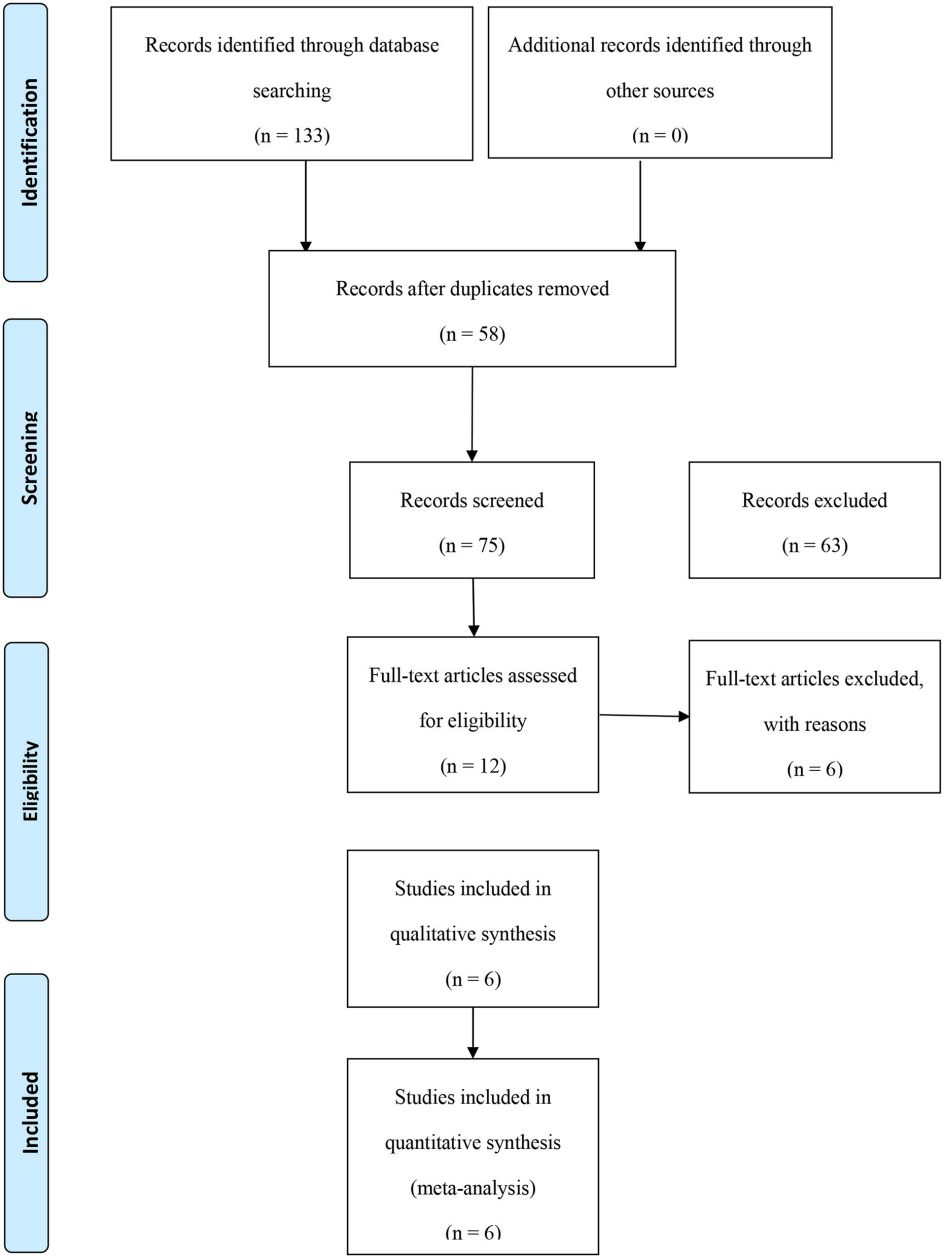
Flow chart of the study selection process.

All of the 322 researches were screened, among them, 12 studies (12–23) involving 35346 PC patients were eligible and were included in our meta-analysis. The process of selecting studies is shown in Figure 1.

The baseline of included studies and the characteristics of PC patients were presented in Table 1. The research types of included studies are cohort studies. Patients who were diagnosed with PC were at an advanced or metastatic stage. Both type 1 diabetes mellitus (T1DM) and type 2 diabetes mellitus (T2DM) patients were included in our meta-analysis. All of these included patients accepted the surgery and metformin treatment. Each of the 12 included trails had calculated the result of a NOS score for each included study which was more than 8 and this presents high methodological quality for this meta-analysis.

**Table 1:** The characteristics of included studies.

**References**	**Country**	**Ethnicity**	**Study design**	**Cancer stage**	**Sample size(N)**	**Surgery strategy**
Lee et al. (13)	Korea	Asian	cohort	I–IV	237	Pancreatectomy
Ambe et al. (12)	USA	Caucasian	cohort	I–II	44	Whipple (18.2%), Non-Whipple (81.8%)
Amin et al. (14)	USA	Caucasian	cohort	I–IV	1,916	Cancer-directed surgery
Chaiteerakij et al. (15)	USA	Caucasian	cohort	I–IV	980	Pancreatectomy
Sadeghi et al. (16)	USA	Caucasian	cohort	I–IV	302	Pancreatectomy
Cerullo et al. (17)	USA	Caucasian	cohort	I–II	3,393	Pancreaticoduodenectomy (60%), partial/distal pancreatectomy (35.7%), total pancreatectomy (4.3%)
Kozak et al. (18)	USA	Caucasian	cohort	I–IV	115	Pancreatectomy
Toomey. et al. (19)	USA	Caucasian	cohort	I–II	414	Pancreatectomy
Beg et al. (20)	USA	Caucasian	cohort	I–IV	13,702	Pancreatectomy
Jang et al. (21)	Korea	Asian	cohort	I–II	764	Whipple/PPPD, distal pancreatectomy
Frouws et al. (22)	Netherland	Caucasian	cohort	I–IV	907	Pancreatectomy
E et al. (23)	USA	Caucasian	cohort	I–IV	12,572	Pancreatectomy

### Overall Survival of Metformin for Pancreatic Cancer Underwent Pancreatectomy

**Figure 2:**
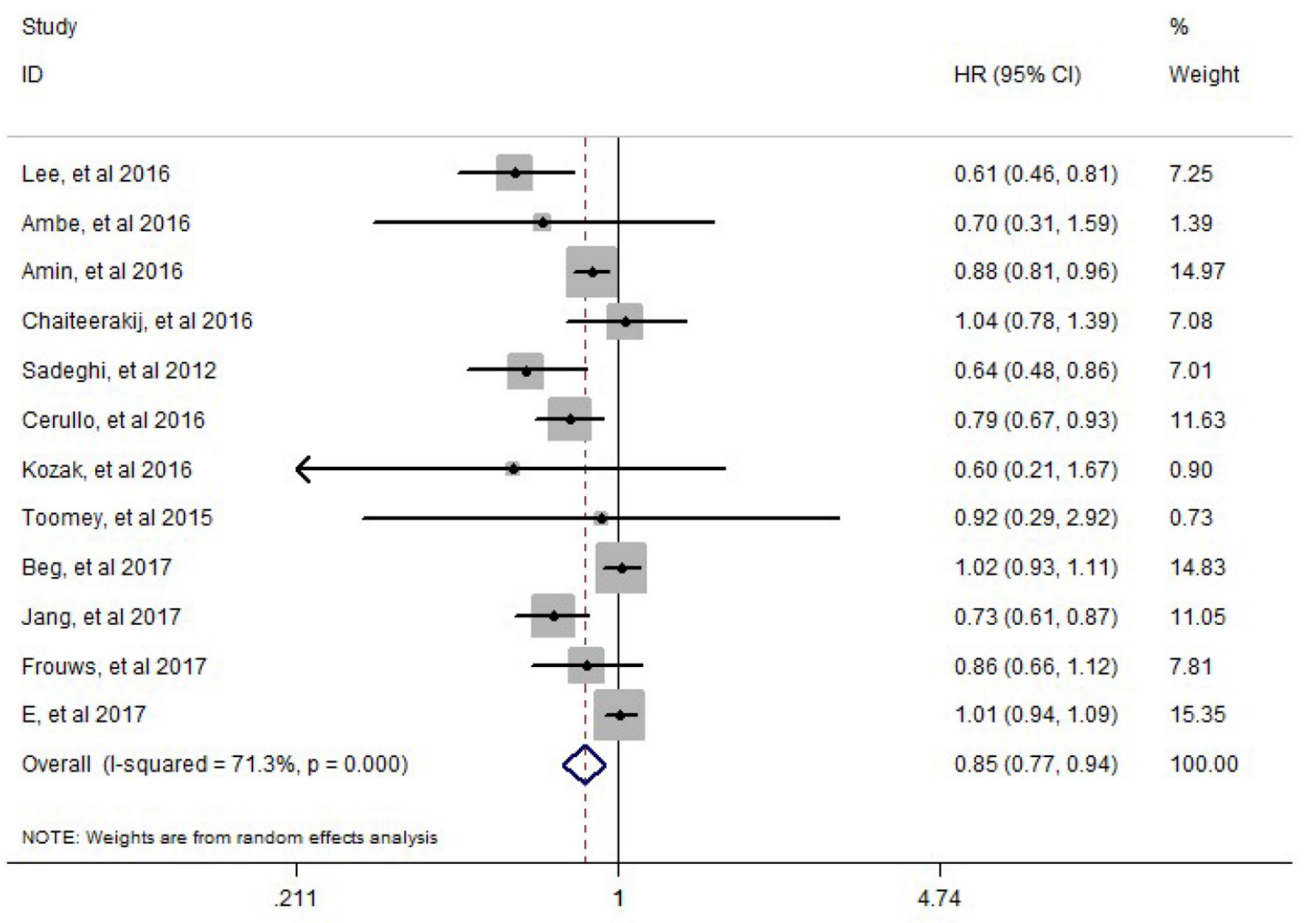
Meta-analysis results of the effect of metformin therapy on the overall survival of pancreatic cancer patients who underwent pancreatectomy.

HR and its 95%CI of overall survival were reported in all included studies. There is inter-study heterogeneity between included studies (*I*^2^= 71.3%, *P* = 0.000). The random-effect model was adopted to perform the meta-analysis, results of which showed that there is a significant difference in the overall survival (HR = 0.85, 95%CI: 0.77–0.94, *P* = 0.002) between PC patients treated with metformin who underwent pancreatectomy and PC patients treated without metformin who underwent pancreatectomy (Figure 2).

### Subgroup Analyses

Subgroup analyses showed Caucasians (HR = 0.903, 95% CI = 0.825–0.940, *P* = 0.008) and Asian (HR = 0.691, 95% CI = 0.588–0.813, *P* = 0.001) PC patients have a significantly reduced risk of death for metformin users. Subgroup analyses also showed a survival benefit for PC patients at stage I-II (HR = 0.762, 95% CI = 0.677–0.858, *P* = 0.0001).

### Sensitivity Analyses

**Figure 3:**
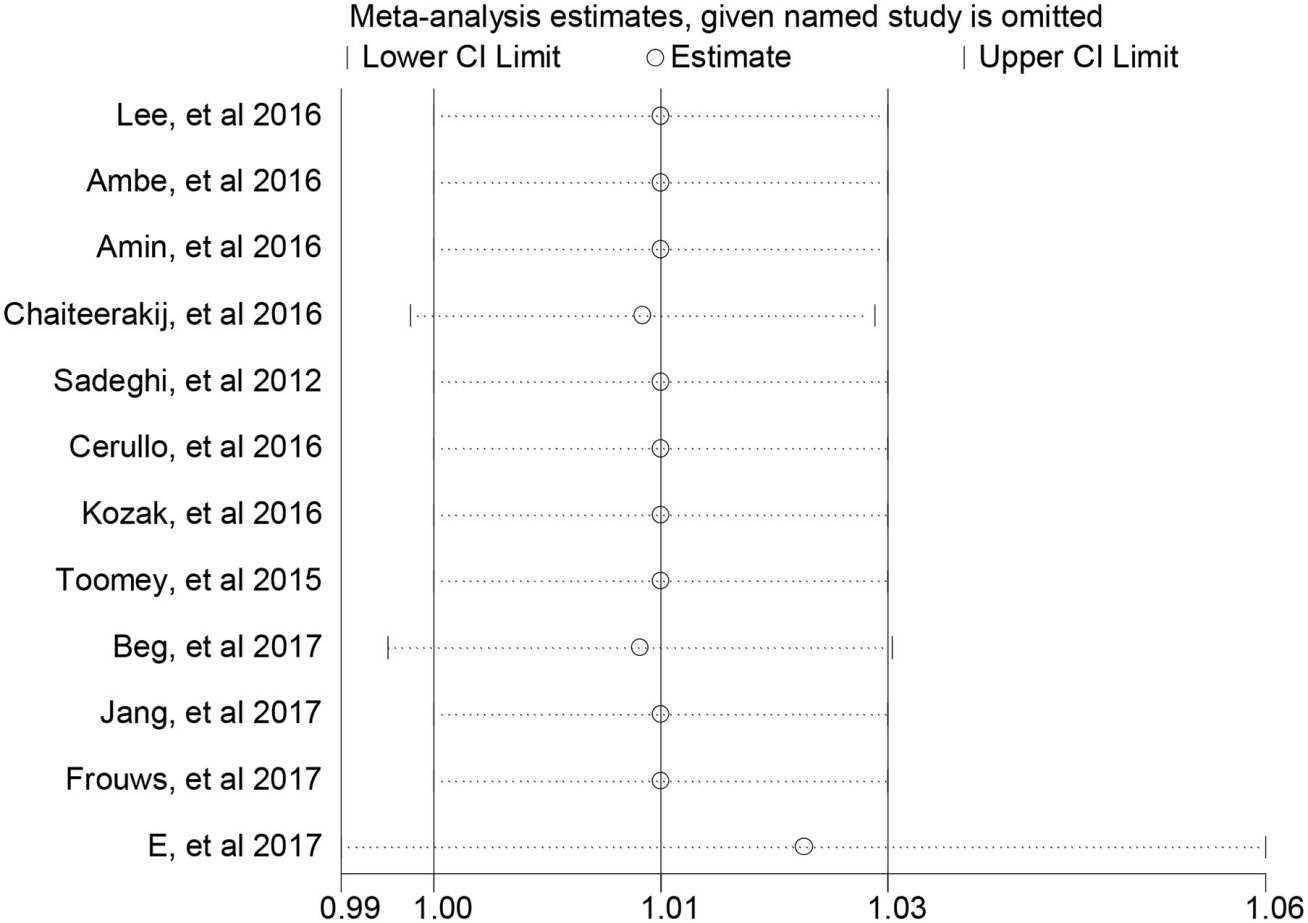
Sensitivity analyses of meta-analysis for overall survival.

By excluding any specific study, we found no substantial alteration among all included studies (Figure 3).

## Discussion

Currently, with the development of treatment strategies for pancreatic cancer including surgery, radiotherapy, chemotherapy, chemoradiotherapy, gene therapy and new target therapeutic, patients with PC were well-treated (24). However, many patients were diagnosed in an advanced or metastatic stage. Distal pancreatectomy, pancreaticoduodenectomy and total pancreatectomy were regarded as the curative surgical treatments for PC patients (25).

The result of this meta-analysis showed that there is a significant difference in overall survival (HR = 0.85, 95%CI: 0.77–0.94, *P* = 0.002) between PC patients treated with metformin who underwent pancreatectomy and PC patients treated without metformin who underwent pancreatectomy. Subgroup analyses showed Caucasians (HR = 0.903, 95% CI = 0.825–0.940, *P* = 0.008) and Asian (HR = 0.691, 95% CI = 0.588–0.813, *P* = 0.001) PC patients have a significantly reduced risk of death for metformin users. Subgroup analyses also showed a survival benefit for PC patients at stage I-II (HR = 0.762, 95% CI = 0.677–0.858, *P* = 0.0001). The previous meta-analyses (26) also showed the same survival benefit from metformin for PC patients. A meta-analysis with four studies involving 1,429 PC patients demonstrated that metformin use can improve the prognosis for PC patients (HR = 0.80, 95% CI = 0.62–1.03) (27). Moreover, a study also showed that metformin use can improve the outcomes of survival for cancer patients such as colorectal cancer, breast cancer, and ovarian cancer (28).

Limitations exist in this meta-analysis: (1) studies we included were all retrospective research, which may influence our meta-analysis. (2) the status of diabetes mellitus was not reported clearly in the included studies and a lack of relevant information about the use of metformin. (3) other important factors such as adverse events, tobacco use, cytotoxicity, which may result in the result of overall survival were not mentioned in included studies.

## Conclusion

Metformin use is associated with survival benefit for PC patients who underwent pancreatectomy, which would be a potential drug for the treatment of PC.

## Data Availability Statement

The datasets are available on request. The raw data supporting the conclusions of this article will be made available by the authors, without undue reservation, to any qualified researcher.

## Author Contributions

JZ, YL, and ZJ planed and designed the research. JZ, JM, and BY tested the feasibility of the study. JZ, JM, and LG wrote the manuscript. All authors approved the final manuscript.

## Conflict of Interest

The authors declare that the research was conducted in the absence of any commercial or financial relationships that could be construed as a potential conflict of interest.
